# The role of α-tubulin tyrosination in controlling the structure and function of hippocampal neurons

**DOI:** 10.3389/fnmol.2022.931859

**Published:** 2022-10-19

**Authors:** Shirin Hosseini, Marco van Ham, Christian Erck, Martin Korte, Kristin Michaelsen-Preusse

**Affiliations:** ^1^Department of Cellular Neurobiology, Zoological Institute, Technische Universität Braunschweig, Braunschweig, Germany; ^2^Research Group Neuroinflammation and Neurodegeneration, Helmholtz Centre for Infection Research, Braunschweig, Germany; ^3^Research Group Cellular Proteome Research, Helmholtz Centre for Infection Research, Braunschweig, Germany

**Keywords:** microtubules, tubulin-tyrosine ligase, dendritic spine, synaptic plasticity, memory

## Abstract

Microtubules (MTs) are central components of the neuronal cytoskeleton and play a critical role in CNS integrity, function, and plasticity. Neuronal MTs are diverse due to extensive post-translational modifications (PTMs), particularly detyrosination/tyrosination, in which the C-terminal tyrosine of α-tubulin is cyclically removed by a carboxypeptidase and reattached by a tubulin-tyrosine ligase (TTL). The detyrosination/tyrosination cycle of MTs has been shown to be an important regulator of MT dynamics in neurons. TTL-null mice exhibit impaired neuronal organization and die immediately after birth, indicating TTL function is vital to the CNS. However, the detailed cellular role of TTL during development and in the adult brain remains elusive. Here, we demonstrate that conditional deletion of TTL in the neocortex and hippocampus during network development results in a pathophysiological phenotype defined by incomplete development of the corpus callosum and anterior commissures due to axonal growth arrest. TTL loss was also associated with a deficit in spatial learning, impaired synaptic plasticity, and reduced number of spines in hippocampal neurons, suggesting that TTL also plays a critical role in hippocampal network development. TTL deletion after postnatal development, specifically in the hippocampus and in cultured hippocampal neurons, led to a loss of spines and impaired spine structural plasticity. This indicates a novel and important function of TTL for synaptic plasticity in the adult brain. In conclusion, this study reveals the importance of α-tubulin tyrosination, which defines the dynamics of MTs, in controlling proper network formation and suggests TTL-mediated tyrosination as a new key determinant of synaptic plasticity in the adult brain.

## Introduction

Microtubules (MTs) are part of the cytoskeleton involved in a variety of cellular functions, including cell division, intracellular transport, and cell shape. They form bundles that are particularly prominent in neurons. Since neurons are differentiated cells, the organization of MTs is critically important for the elaborate architecture of axons and dendrites (Dent and Baas, 2014). They play, for instance, critical roles in the formation and maintenance of axonal growth cones during development and in regeneration after injury (Bradke et al., 2012). In neurons, MTs consist of both highly dynamic and stable subpopulations that correlate with neuronal subcompartments. In the axon, a higher percentage of the total microtubule mass is stable compared to the situation in the dendrite (Baas et al., 2016). Beyond this general role in supporting neurite integrity and organelle transport, MTs may be even more actively involved in signal transduction (Dent and Baas, 2014). At the plus-ends of highly dynamic MTs, end-binding proteins (+TIPs) are associated with signaling cascades important for neuronal structure and plasticity (Baas et al., 2016).

Dynamic MTs substantially contribute to modulating synaptic structure and function. In the presynaptic compartment, dynamic MTs control neurotransmission by promoting the transport of synaptic vesicles for neurotransmitter release (Qu et al., 2019). At the postsynapse, which is predominantly localized at dendritic spines in cortical brain regions, the major cytoskeletal element is F-actin (Hotulainen and Hoogenraad, 2010), yet a structural and functional interplay between dendritic MTs and the actin cytoskeleton within spines has been described (Jaworski et al., 2009). Dynamic MTs frequently invade dendritic spines and are important for spinogenesis, synaptic targeting of NMDA receptors, and even synaptic plasticity (Gu et al., 2008; Kapitein et al., 2011; Martel et al., 2016). This is supported by the fact that blocking dynamic MTs by low doses of nocodazole impairs long-term potentiation (LTP) (Jaworski et al., 2009). Consistent with these findings, the transient accumulation of dynamic MTs in hippocampal dendritic spines is necessary for learning and memory processes in contextual fear conditioning, NMDA-dependent memory tasks, and memory discrimination tasks (Uchida et al., 2014; Martel et al., 2016). Although the general importance of dynamic MTs for synaptic function and plasticity has been demonstrated, the detailed molecular mechanisms by which dynamic MTs are regulated in neurons remain mostly elusive.

Post-translational modifications (PTMs) of tubulin subunits increase their diversity and provide potential mechanisms for the functional specialization of MTs (Wloga and Gaertig, 2010). Conserved tubulin PTMs include detyrosination/tyrosination, acetylation, glycylation, and glutamylation (Janke and Bulinski, 2011). Post-translational detyrosination/tyrosination has been described mainly in the nervous tissue. In this process, tubulin at the carboxyl-terminus of the alpha-tubulin subunit can be modified by the cyclic release and re-addition of the tyrosine residue. Two enzymes are involved in these reactions: tubulin tyrosine carboxypeptidase (Aillaud et al., 2017; Nieuwenhuis et al., 2017) and tubulin tyrosine ligase (TTL) (Barra et al., 1988), respectively.

We have previously shown that conventional deletion of TTL blocks neurite extension *in vivo* and arrests neuronal development, leading to perinatal death (Erck et al., 2005). TTL null embryos showed a disrupted cortico-thalamic loop and impaired layering of the neocortex (Erck et al., 2005). In contrast, *in vitro* cultured TTL null hippocampal neurons showed accelerated neurite and axonal growth compared with wild-type neurons (Erck et al., 2005). These results were confirmed by TTL knockdown experiments in cultured rat hippocampal neurons, showing a 40% increase in neurite growth and, in contrast, a 35% decrease in neurite growth after TTL overexpression (Prota et al., 2013). Apparently, suppression of TTL *in vivo* disrupted mechanisms important for the control of neurite outgrowth, but not neurite outgrowth *per se* (Erck et al., 2005). Thus, the evolutionarily highly conserved tubulin tyrosination cycle in neurons most likely plays a crucial role in neurite formation and microtubule stabilization. Whereas, detyrosinated MTs tend to be rather stable, tyrosination renders them more dynamic (Webster et al., 1987), suggesting that post-translational detyrosination/tyrosination of tubulin might represent a mechanism for modulating MT dynamics not only during neurite growth and stabilization but, beyond this, also in synaptic plasticity. Consistent with this, recent evidence has suggested that modulation of MT dynamics in the adult brain may also be involved in learning processes and memory formation and may be impaired in neurodegenerative diseases, such as Alzheimer’s disease (Dent, 2017).

We, therefore, aimed to investigate how MT dynamics can modulate brain development and control neuronal function in adult mice using conditional deletion of TTL in different mouse lines. Our results suggest that tyrosination of α-tubulin plays a critical role not only during brain development, particularly in cortical regions, but also in functional processes and structural plasticity in mature hippocampal neurons. Knowledge about the detailed role of the tubulin tyrosination cycle controlling MT dynamics during neuronal plasticity could be used in the future to develop new treatment strategies for neurodegenerative diseases.

## Materials and methods

### Mice

Mice were housed under specific pathogen-free conditions in the animal facility of the Helmholtz Center for Infection Research (Braunschweig, Germany). Animal care and all procedures were performed in accordance with institutional, state, and federal guidelines. The targeting construct was generated using the Gene Bridges’ Red/ET recombination technique and standard protocols (Gene Bridges GmbH, Heidelberg, Germany). Briefly, a loxP site was inserted 494bp upstream of exon 4 using a PGK-neo-loxP template and two primers with a 50-bp homolog for this region. Subsequently, the PCR product was inserted into a 7,130-bp genomic fragment by Red/ET recombination. Subsequently, the selection marker was removed by transformation in 294-Cre *Escherichia coli*. The second loxP site was then inserted 416 bp downstream of exon 5 using PGK-neo-FRT. After Red/ET recombination, the targeting vector was sequenced and electroporated into embryonic stem (ES) cells. Selected positive ES cell clones were transiently transfected with FLP recombinase to delete the selection marker. One ES cell clone was injected into C57BL/6 blastocysts, resulting in four chimeric males that were used for breeding with C57BL/6 females to allow germline transmission. Heterozygous progenies were backcrossed to C57BL/6 and finally crossed to NexCre (Goebbels et al., 2006) and CaMKIIα-Cre line T29-1 mice (Tsien et al., 1996).

The following primers were selected for genotyping the mice.

**Table:** 

TTL-loxed allele:	Forward	CCAGAGGCCCGGTTTCCCAGG
	Reverse	CTCTTCTAAGATGATGCCCTATGG
TTL-wt allele:	Forward	CCAGAGGCCCGGTTTCCCAGG
	Reverse	GAACTGATGGCGAGCTCAGACC
TTL-deleted allele:	Forward	CCAGAGGCCCGGTTTCCCAGG
	Reverse	GAGCTAGCTGCCCTGCTAGAGC
Cre-recombinase:	Forward	ACGACCAGGTGACAGCAATG
	Reverse	CTCGACCAGTTTAGTTACCC

Genotyping of conventional TTL knockout mice was performed as previously described (Erck et al., 2003, 2005).

The experiments performed with mice were approved according to animal welfare law in Germany. All protocols used in this project have been reviewed and approved by the local committees at TU Braunschweig and the authorities (LAVES, Oldenburg, Germany 33.19-42502-04-20/3498) according to the national guidelines of the animal welfare law in Germany [“Tierschutzgesetz in der Fassung der Bekanntmachung vom 18. Mai 2006 (BGBl. I S. 1206, 1313), das zuletzt durch Artikel 20 des Gesetzes vom 9. Dezember 2010 (BGBl. I S. 1934) geändert worden ist.”].

### Western blotting

To quantify the expression of tyrosinated tubulin in the cortical part and the PSD-95 level as a marker of the excitatory postsynaptic compartment in the cortex and hippocampus of 10-week-old TTL^lox/lox/NexCre+^ mice and control littermates, Western immunoblotting was performed.

In brief, the mice were deeply anesthetized with CO_2_ and decapitated. Tissue was isolated and lysed in a buffer containing 25-mM Tris pH 7.5, 1-mM EDTA, 1-mM EGTA, and 1% SDS. Ten-microgram total lysates were analyzed according to standard protocols as previously described (Hosseini et al., 2020). Membranes were incubated overnight at 4°C with an anti-Tyr-alpha-tubulin antibody (1:500, Synaptic Systems Cat# 302 117, RRID:AB_2620047), an anti-alpha-tubulin antibody (1:2,000, Synaptic Systems Cat# 302 411, RRID:AB_2631215), or an anti-PSD-95 antibody (1:1,000, Synaptic Systems Cat# 124 011, RRID:AB_10804286). After washing, the membranes were incubated for 1 h at room temperature with the appropriate secondary peroxidase-conjugated antibodies from Jackson ImmunoResearch. Immunoblots were analyzed by luminometry using a cooled charge-coupled camera (Fuji, Japan) and AIDA software (Raytest, Straubenhault, Germany).

### Immunohistochemistry and DiI staining

Ten-week-old TTL^lox/lox/NexCre+^ mice and control littermates were perfused first with physiological saline containing heparin and then with phosphate-buffered 4% paraformaldehyde (PFA). The brains were carefully dissected and postfixed overnight with the same fixative at 4°C. Fifty-μm-thick coronal and sagittal sections were cut using an HM650V Vibratome (Thermo Scientific, USA). Sections were collected in 0.1-M Tris-buffered saline pH 7.4 (TBS), containing sodium azide and stored at 4°C until further use. For immunohistochemistry, free-floating sections were washed with TBS and blocked with TBS containing 5% normal donkey serum and 0.3% Triton X-100 for 1 h at room temperature. Sections were then incubated overnight at room temperature with primary antibodies diluted in a blocking buffer, including an anti-Tyr-alpha-tubulin antibody (1:500, Synaptic Systems Cat# 302 117, RRID:AB_2620047), an anti-Cre recombinase antibody (1:500, Synaptic Systems Cat# 257 003, RRID:AB_2619968), an anti-MAP-2 antibody (1:500, Synaptic Systems Cat# 188 011, RRID:AB_2147096), and an anti-parvalbumin antibody (1:500, Synaptic Systems Cat# 195 011, RRID:AB_2619882). After washing with TBS, sections were incubated with appropriate fluorophore- or peroxidase-conjugated secondary antibodies (Dianova or Molecular Probes) diluted in TBS containing 2% bovine serum albumin (BSA) for 1 h. Peroxidase-conjugated secondary antibodies were detected by DAB staining (Sigma Aldrich, USA). Cell nuclei were counterstained with DAPI (Sigma Aldrich, USA). Finally, the sections were washed extensively with TBS, rinsed briefly with distilled water, air dried, and mounted on glass slides with Entellan™ in toluene (Merck Millipore, Germany). Images were acquired using a 510 Meta confocal laser scanning microscope (Zeiss, Germany) or an ECLIPSE Ti-E-inverted microscope (Nikon, Japan) with a DS2-MBWc camera (Nikon, Japan). Bright-field images for a brain wave analysis were acquired with an SZX12 microscope (Olympus, Japan) and a DP26 camera (Olympus, Japan). Data acquisition and analysis were performed using LSM software (Zeiss, Germany), NIS elements (Nikon, Japan), or Photoshop.

**FIGURE 1:**
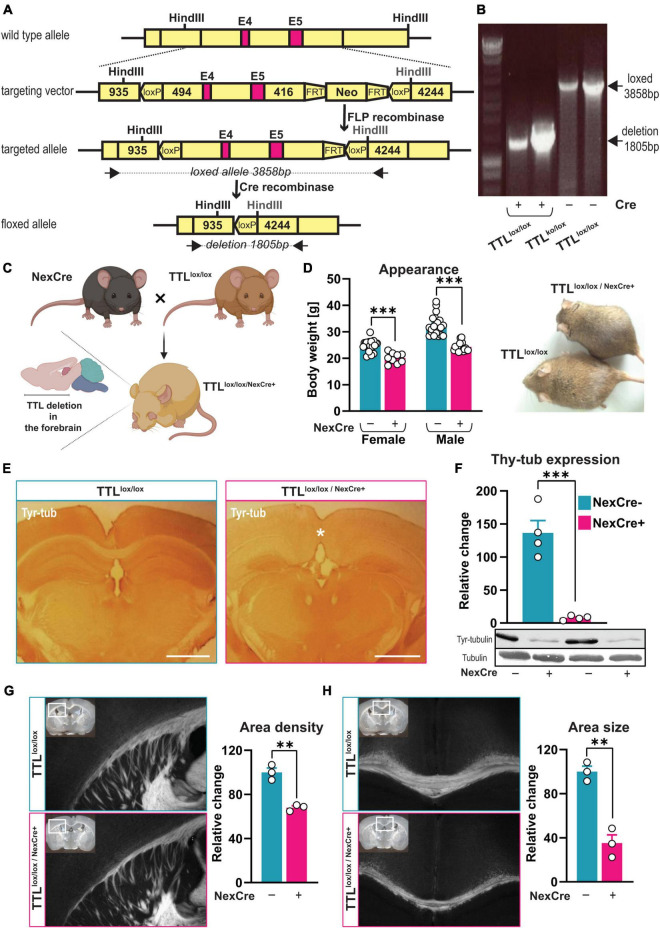
Generation of a TTL conditional knock-out mouse line. **(A)** A schematic overview of the targeted allele and the resulting floxed allele. Exons 4 and 5 (E4 and E5) are flanked with loxP sites, and the neomycin cassette (Neo) was removed by FLP recombinase. The floxed allele shows the resulting genomic DNA structure after Cre-recombinase deletion of Exons 4 and 5, leaving one loxP site. Black arrows indicate the oligos used [see panel **(B)**]. The indicated *Hin*dIII sites were used to determine the genomic structures. FRT: FLP-recognition target. **(B)** Representative image showing the genomic deletion of the target allele. Genomic DNA was isolated from mouse embryonic fibroblasts (MEFs) transduced to express different amounts of Cre-recombinase/EGFP fusion protein (Cre+). PCR was used to detect the deleted floxed allele [1,805 base pairs (bp)]. The 3,858-bp band corresponds to the PCR product of the non-targeted loxP/loxP allele in non-transduced control cells (Cre-). **(C)** Schematic representation of TTL^lox/lox/NexCre+^ mouse generation (the figure was created using BioRender.com). **(D)** 10-week-old female and male TTL^lox/lox/NexCre+^ mice displayed lower body weight compared to their control littermates (*n* = 9–21, unpaired *t*-test, *p* < 0.001). **(E)** An overview of the decreased levels of tyrosinated α-tubulin in the neocortex and hippocampus of TTL^lox/lox/NexCre+^ mice. Brain sections were immunostained with an antibody against tyrosinated α-tubulin (scale bar = 1 mm). **(F)** Western blot analysis of cortical extracts from the 10-week-old mice showed an approximately 95% reduction in tyrosinated α-tubulin in the TTL^lox/lox/NexCre+^ mice compared with the control mice. The relative intensities of the signals were measured by densitometry and normalized to total α-tubulin (*n* = 4). Representative immunoblots using antibodies against tyrosinated α-tubulin and total α-tubulin are shown. **(G,H)** Reduced development of cortico-thalamic neuron projections **(G)** and corpus callosum **(H)** in brains of TTL^lox/lox/NexCre+^ mice. Brain sections from the 10-week-old TTL^lox/lox/NexCre+^ mice and the control littermates were stained using the DiI/SWITCH method. Representative images are shown (overviews are shown as insets, and white boxes indicate a higher magnification area). Measurements of the relative area density of cortico-thalamic neuronal projections and the area size of the corpus callosum (with Fiji) revealed a 32% reduction in the density of cortico-thalamic neuronal projections **(G)** and a 65% reduction in the size of the corpus callosum in the NexCre + mice compared with the control [**(H)**, see also the asterisk in panel **(E)**, the right panel, *n* = 3]. Data are presented as mean ± SEM, ^**^p < 0.01 and ^***^p < 0.001.

For DiI staining, brains from the 10-week-old mice were isolated and fixed as described above. After fixation, the brains were washed with PBS. Brain sections (50 μm) were prepared using an HM650V Vibratome (Fisher Scientific™, USA). A series of sections was stained with DiI (1,1’-dioctadecyl 3,3,3’-tetramethylindocarbocyanine perchlorate, Molecular Probes) using the SWITCH (system-wide control of interaction time and kinetics of chemicals) technique (Murray et al., 2015). The sections were imaged using an ECLIPSE Ti-E-inverted microscope (Nikon, Japan) with a DS2-MBWc camera (Nikon, Japan). Image analysis was performed using Fiji software (BioVoxxel). To quantify the area density of the cortico-thalamic neuronal projections, the corrected total fluorescence intensity was measured in each region of interest (ROI), as shown in the representative images (Figure 1G). To quantify the size of the corpus callosum, the area of the medial corpus callosum and lateral corpus callosum (as shown in the representative images, Figure 1H), defined using polygon selection, was measured. Data were normalized to the control group. All slides were coded, and analysis was performed blindly.

### Golgi-Cox staining

To investigate the density of dendritic spines, Golgi-Cox staining was performed. For this purpose, the left hemispheres of 4–5-month-old TTL^lox/lox/NexCre+^ and TTL^lox/lox/CaMKIIα–Cre+^ mice and control littermates were incubated with the FD rapid Golgi stain kit (FD NeuroTechnologies, Inc., Columbia, SC, USA) according to the manufacturer’s protocol. Hemispheres were then blocked in 2% agar, and 200-μm-thick coronal sections were cut using a vibratome (Leica VT 1,000 S, Germany) and mounted on gelatin-coated glass slides. The sections were then processed for signal development before dehydration through graded alcohols and mounting with Permount (Fisher Scientific, USA). To examine the morphology of hippocampal neurons in Golgi-Cox-stained sections, second- or third-order branching of apical dendrites in CA1 and CA3 and dendrites in the superior and inferior dentate gyrus subregions of the hippocampus (10 cells per animal) was analyzed three-dimensionally (Z-stack thickness of 0.5 μm) using an Axioplan 2 imaging microscope (Zeiss, Germany) equipped with a 63X (N.A. 1) oil objective and a digital camera (AxioCamMRm, Zeiss, Germany). For all selected neurons, spine density per micrometer of dendrites was calculated using the Fiji software (BioVoxxel) on the segments of dendritic branches longer than 60–70 μm that were at least 50 μm from the soma. The total number of spines along the segments of dendritic branches was manually counted by an investigator who was blinded to all experimental groups.

### Behavioral experiments

For behavioral assessment, 4–5-month-old TTL^lox/lox/NexCre+^, TTL^lox/lox/CaMKIIα–Cre+^ mice, and control littermates were used. All behavioral experiments were performed at the same time of the day during the light period in dim light between 9:00 a.m. and 4:00 p.m. by a blinded experimenter for all groups.

#### Open-field test

In this study, spontaneous locomotor activity, readiness to explore, and initial screening of anxiety-like behavior were examined with the open-field test, as described previously (Walsh and Cummins, 1976). Briefly, the mice were placed on one side of a white PVC arena (50 cm × 50 cm × 50 cm) for 5 min. The central (30 cm × 30 cm × 30 cm) and core (10 cm × 10 cm × 10 cm) zones of the arena were defined as the middle section. Between each experimental run, the apparatus was completely cleaned with ethanol 70% to reduce odor perception. Movement data, including total distance traveled, average speed, frequency of core visits, and percentage of time the mice spent in the periphery and center zones of the arena, were collected using Video Mot 2 (TSE Systems GmbH, Bad Homburg, Germany) or ANY-maze (Stoelting, USA) behavioral tracking software.

#### Morris water maze test

To investigate the cognitive behavior of the mice, spatial learning and memory formation were examined using the Morris water maze paradigm. Spatial learning in the Morris water maze is a hippocampus-dependent task that requires animals to find a hidden platform based on visual cues around the pool (Morris, 1984). The maze consisted of a circular pool with a diameter of 160 cm filled with 19–20°C opaque water (titanium dioxide, Euro OTC Pharma). The transparent platform was 10 cm in diameter and hidden 1 cm below the water surface. Before training, a visible platform task was performed as pre-training to ensure that swimming ability and visual acuity were intact in both experimental groups. In addition, this phase was important to allow the animals to become accustomed to the test situation. During this phase, the animals had two trials (maximum 60 s each) per day for three consecutive days to reach the visible platform, changing position during the trials (data not shown). Training was then conducted for 8 days in the Morris water maze, with the invisible platform located in the northwest (NW) quadrant. Each day, the animals were placed in the water for four trials, with different starting points (SW, S, E, and NE) randomly assigned to a 5-min interval between trials. The animals were allowed to swim freely for 60 s or until they reached the platform. Otherwise, they were led to the platform and allowed to sit on it for 20 s. For the qualitative aspects of spatial memory formation during the 8-day acquisition training, the trace map for finding the platform was analyzed as a search strategy. Over time, the mice switched from egocentric strategies independent of the hippocampus, such as scanning, characterized by >75% surface coverage; chaining, characterized by >75% time in a donut-shaped annulus zone; and random swimming characterized by >80% surface coverage, to an allocentric-directed swimming search strategy characterized by >50% time in Whishaw’s corridors (a 40° target corridor) that depends on the hippocampus (Garthe et al., 2009; Garthe and Kempermann, 2013). To assess memory retrieval, three probe trial tests were conducted on the 3rd and 6th days of the acquisition training before the four trials of the training began that day. Another test of reference memory was conducted 24 h after the last day of the acquisition training (Day 9). During the probe trials, the platform was removed and the animals were allowed to swim freely for 45 s.

All data, including escape latency (time to reach the platform), percentage of time spent in the four quadrants of the pool, percentage of time spent in the peripheral (thigmotaxis), donut-shaped annulus (chaining), central circle (scanning), and Whishaw’s corridors (direct search) of the pool, were recorded using Video Mot 2 (TSE Systems GmbH, Bad Homburg, Germany) or ANY-maze (Stoelting, USA) behavioral tracking software.

### Electrophysiological experiments

Hippocampi of 4–5-month-old mice were dissected, and 400-μm-thick transverse acute hippocampal slices were cut using a manual tissue chopper. The slices were then immediately incubated in an interface chamber (Scientific System Design) at 32°C for 2 h and continuously perfused with oxygen-enriched artificial cerebrospinal fluid (aCSF, pH 7.2) at a flow rate of 0.5 ml/min. The aCSF contained the following chemicals (in mM): 124 NaCl, 4.9 KCl, 1.2 KH_2_PO_4_, 2. MgSO_4_, 2. CaCl_2_, 24.6 NaHCO_3_, 10 D-glucose equilibrated with 95% O_2_-5% CO_2_ (32 L/h).

For stimulation, a monopolar, lacquer-coated stainless steel electrode (5 MΩ; AM Systems) was placed in the stratum radiatum of the CA1 region to generate field excitatory postsynaptic potentials (fEPSP) from Schaffer collateral/commissural CA1 synapses. For recording the fEPSP (measured as its initial slope function), an electrode (5 MΩ; AM Systems, USA) was placed in the apical dendritic layer of the CA1 region, and signals were amplified with a differential amplifier (Model 1700, AM Systems). Signals were digitized using a CED 1401 analog-to-digital converter (Cambridge Electronic Design). The slopes of the fEPSPs were monitored during recording.

An input-output curve (afferent stimulation vs. a fEPSP slope) measuring basal synaptic transmission was obtained 2 h after pre-incubation. The intensity of the test stimulation was adjusted so that the slope of the fEPSP was 40% of the maximum fEPSP response. In the TTL^lox/lox/NexCre+^ and the corresponding control group, long-term potentiation (LTP) was induced by a “strong” tetanization protocol (STET), consisting of three stimulus trains of 100 pulses at 100 Hz (stimulus duration of 0.2 ms/polarity, an intertrain interval of 10 min, total of 300 pulses). Four biphasic 0.2-Hz constant current pulses (0.1 ms/polarity) were used for baseline recording. In TTL^lox/lox/CaMKIIα–Cre+^ mice and littermate controls, 20 min after baseline recording, LTP was induced by θ-burst stimulation (TBS) with four bursts at 100 Hz repeated 10 times at an interval of 200 ms. This stimulation was repeated three times at an interval of 10 s. In all electrophysiological experiments, only healthy sections with a stable baseline were included in the data analysis. The slope of fEPSPs was measured over time and normalized to the baseline. Data acquisition and offline analysis were performed with the IntraCell software (version 1.5, LIN, Magdeburg, 2000, Germany).

### Live-cell imaging

To investigate activity-dependent structural plasticity, organotypic hippocampal slice cultures (OHCs) from the TTL^lox/lox^ mice were prepared on postnatal Day 5 and maintained in an Eagle basal medium (Thermo Fisher Scientific, USA), containing 25% horse serum, 1-mM L-glutamine (Sigma Aldrich, USA), 0.5% glucose, and 25% HBSS (Invitrogen, USA), as previously described (Michaelsen-Preusse et al., 2014). After 72 h of incubation, the antimitotic agent was added for 24 h and removed by a 100% media change, followed by weekly media changes of 50%. At DIV 14, OHCs were transfected with pmApple-N1 (for dendritic spine structural analysis) and pCAG-Cre:GFP plasmids (for TTL deletion) by electroporation of single cells as previously described (Michaelsen-Preusse et al., 2014). DNA constructs were electroporated in HBSS (Invitrogen) at RT with 5 V, 1 mA, and a 220-Hz train.

After 4 days, OHCs were incubated in aCSF (as described above) saturated with 95% O_2_ and 5% CO_2_ at 32°C for 20 min in an imaging chamber of the Olympus Fluoview 1000 laser scanning microscope equipped with a UPLFLN 60x water (NA = 1) immersion objective and Olympus Fluoview Ver. 4.0, a software at a constant percentage of laser exposure, with a pixel size of 0.069 μm. The apical dendritic segment of the pyramidal neuron was imaged 20 min before chemical LTP induction (cLTP). Then, cLTP was induced by incubating OHCs in 10-mM glycine (AppliChem, Germany) and 3-μM strychnine (an inhibitory ionotropic glycine receptor blocker) (Sigma Aldrich, USA) in aCSF for 10 min. Then, the cLTP solution was exchanged for aCSF, and the same dendritic segment was imaged again every 20 min for 1 h after stimulation. The density of dendritic spines was analyzed as described above in the Golgi-Cox staining section. In addition, 15 spines along each dendrite were randomly selected for further morphological assessment, and the diameter of the spine head was measured using Fiji software (BioVoxxel) by an investigator who was blinded to all experimental groups.

### Statistical analysis

Data were analyzed and plotted using GraphPad Prism 8 (GraphPad Software, Inc., USA) and presented as mean ± SEM. Differences in body weight, dendritic spine density, spine head diameter, relative changes in intensity and size, total distance traveled, average velocity, and number of core visits were examined with a Student’s *T*-test, whereas a two-way ANOVA test was used for the behavioral and electrophysiological experiments. The Bonferroni multiple comparison correction was used as a *post hoc* test. The minimum significance value was considered as *p* < 0.05. The minimum number of animals in all experiments was calculated *a priori* using G*Power 3.1.9.4 software (Heinrich Heine College, Düsseldorf, Germany). All statistical analyses and *n* of the different experimental groups were reported in the respective results or figure legends.

## Results

### Generation of a tubulin-tyrosine ligase conditional knock-out mouse line

Previous findings have shown that full ablation of TTL leads to perinatal death (Erck et al., 2005). Thus, to investigate the role of α-tubulin tyrosination, specifically in adult brain structure and function, a mouse line was generated that allows tissue- and time-specific deletion of TTL. To this end, a targeting vector was created in which the inserted LoxP sites flanked Exons 4 and 5 (Figure 1A; Erck et al., 2003). Deletion of both exons, which contain parts of the active domain as well as the ATP-binding domain (Szyk et al., 2011), therefore, should result in a truncated and inactive TTL product consisting of 157 amino acid residues. The targeting vector was electroporated into embryonic stem cells (ES), and, as confirmed by PCR and Southern blot analysis, two ES cell clones with the correctly integrated targeting construct were obtained. One clone was injected into C57BL/6 blastocysts, resulting in four chimeric males that were used for breeding to enable germline transmission. Heterozygous progenies were backcrossed with C57BL/6 and finally interbred to obtain homozygous TTL^lox/lox^ mice. The mice were born with a normal Mendelian ratio, were fertile, and had no obvious abnormalities or impaired TTL activity. In addition, the TTL^lox/lox^ mice were crossed with conventional heterozygous TTL^±^ mice (Erck et al., 2005) to obtain TTL^ko/lox^ mice that support efficient deletion of only one loxed allele.

To demonstrate efficient deletion of the *ttl* gene, mouse embryonic fibroblasts (MEFs) were isolated from TTL^lox/lox^ cultivars and transduced to induce expression of a Cre recombinase/EGFP fusion protein. Using deletion-specific PCR on transduced MEFs, a unique PCR product was detected only in Cre recombinase-expressing cells (Figure 1B). Western blot analysis failed to reveal a truncated TTL protein product indicative of unstable mRNA and/or protein products (data not shown). Remarkably, a first attempt to generate a conditional TTL knockout mouse line—with an inserted loxP site 109 base pairs upstream of the ATG codon—showed the same lethal phenotype as observed in conventional TTL knockout mice, even before Cre recombinase-dependent excision (data not shown). This suggests that an intact 5′-flanking region within the *ttl* gene is required for proper TTL expression.

### Mice lacking tubulin-tyrosine ligase in the neocortex and hippocampus are viable, albeit with obvious impairments in brain development

Previously, it has been shown that, in conventional TTL^–/–^ embryos, TTL activity is absent in both cortical and thalamic parts of the developing brain, resulting in severe blockade of ascending and descending neuronal projections (Erck et al., 2005). To study cognitive function in the absence of TTL, a mouse strain expressing Cre recombinase under the control of the *nex* promoter was used to drive TTL deletion only in the forebrain (Figure 1C; Goebbels et al., 2006). In a previous study, the NexCre expression pattern was analyzed using different lacZ reporter mice. The strongest Cre activity was observed in the neocortex and hippocampus, starting around embryonic Day 11.5. Cre-mediated recombination marked pyramidal neurons and mossy fibers of the dentate gyrus but was absent in proliferating neural precursors of the ventricular zone, interneurons, oligodendrocytes, and astrocytes (Goebbels et al., 2006).

In contrast to the conventional TTL knockout mice, the TTL^lox/lox/NexCre+^ mice were born in a normal Mendelian ratio, and were healthy and fertile. Yet, compared with control littermates (males: 32.45 ± 0.82 g, females: 24.81 ± 0.52 g), adult mice were smaller and had significantly lower body weights [males: 24.28 ± 0.39 g (unpaired *t*-test: *t* = 8.48, df = 37, *p* < 0.001), females: 20.08 ± 0.65 g (unpaired *t*-test: *t* = 5.40, df = 25, *p* < 0.001), Figure 1D].

The efficiency of TTL deletion in Cre recombinase-expressing cells was assessed by immunostaining of tyrosinated α-tubulin. Ten-week-old control mice (TTL^lox/lox/NexCre–^) showed the expected general immunoreactivity throughout the brain (Figure 1E, the left panel). In contrast, age-matched TTL^lox/lox/NexCre+^ mice exhibited approximately 95% reduced levels of tyrosinated α-tubulin in the neocortex and pyramidal neurons of the hippocampus (Figure 1E, the right panel). This was also confirmed by Western blotting of cortical extracts from the TTL^lox/lox/NexCre+^ mice (NexCre-: 136.5 ± 18.70%, NexCre+: 7.97 ± 1.67%, unpaired *t*-test: *t* = 6.84, df = 6, *p* < 0.001, Figure 1F).

In addition, double immunostaining with antibodies directed against Cre recombinase and tyrosinated tubulin (Supplementary Figures 1A,B) revealed the expected Cre recombinase expression pattern in the pyramidal cell layer of the hippocampus but not in granule cells in the dentate gyrus, as well as general immunostaining with tyrosinated α-tubulin in 10-week-old control mice (TTL^wt/lox/NexCre+^). The same Cre expression pattern was found in the TTL^lox/lox/NexCre+^ mice, but tyrosinated tubulin immunostaining was largely absent. Only a few cells, most likely NexCre-negative interneurons and astrocytes, were immunopositive for tyrosinated α-tubulin. High levels of tyrosinated α-tubulin were observed in the thalamus, where there are actually no Cre recombinase-expressing cells (Supplementary Figure 1A, the asterisk). Normal levels of tyrosinated α-tubulin were detected in the granule cells of the dentate gyrus (Supplementary Figure 1A, the arrow), most likely due to transient expression of Cre recombinase over time in this area (Goebbels et al., 2006). The described absence of Cre recombinase expression in interneurons of TTL^lox/lox/NexCre+^ mice was supported by the presence of parvalbumin-positive neurons with strong immunoreactivity for tyrosinated α-tubulin (Supplementary Figure 1C).

To test whether projecting neurons from the cortex in TTL^lox/lox/NexCre+^ mice were able to find their targets in other brain regions and make contacts, brain slices were labeled with DiI, a fluorescent lipophilic cationic indocarbocyanine dye, using the SWITCH technique (Murray et al., 2015) to visualize neuronal trajectories. In contrast to conventional TTL-deficient embryos, high numbers of projecting axons exiting the cortex were observed in the 10-week-old TTL^lox/lox/NexCre+^ mice, albeit with an approximately 32% reduction in fiber area density (67.99 ± 1.68%) compared with control littermates (100 ± 3.94%, unpaired *t*-test: t = 7.48, df = 4, *p* = 0.0017, Figure 1G).

The development of the corpus callosum begins with the growth of pioneer axons located in the cingulate cortex and crossing the midline at E15.5, followed by neocortical axons crossing at E16.5. The vast majority of axons forming the corpus callosum originate from the neocortical layers II, III, and V (Richards et al., 2004). To examine the integrity of the fiber tracts, coronal sections from the 10-week-old mice were analyzed using the DiI/SWITCH technique as described above. In the TTL^lox/lox/NexCre+^ mice, the corpus callosum exhibited hypoplasia, a 65% reduction in the area size of the fiber tracts (NexCre-: 100 ± 4.91%, NexCre+: 35.15 ± 7.55%, unpaired *t*-test: *t* = 7.19, df = 4, *p* = 0.002, Figure 1H). In addition, rostro-caudal reduction of the corpus callosum was evident from the absence of midline-crossing fibers at the splenium (see asterisks in Figure 1E and Supplementary Figure 2A). This indicates a developmental arrest of the corpus callosum at Day E16-17 in TTL^lox/lox/NexCre+^ mice.

In addition, analysis of serial slices revealed a reduction in hippocampal commissure (HC) size leading to midline gap formation in the TTL^lox/lox/NexCre+^ mice (Supplementary Figures 2B,C).

To reveal details about the late onset of developmental differences between control mice and TTL^lox/lox/NexCre+^ mice, we analyzed newborn pups to follow the time course of downregulation of tyrosinated α-tubulin. Western blot analysis of cortex lysates from P3 and P5-aged pups showed no apparent reduction in tyrosinated α-tubulin in the TTL^lox/lox/NexCre+^ mice. Starting at P7 (Supplementary Figure 2D) and even more markedly in the adult mice (Figure 1F), the amount of tyrosinated α-tubulin decreased. Although Cre recombinase expression began at E11.5, resulting in deletion of the TTL gene, a complete loss of tyrosinated α-tubulin was observed much later potentially, indicating a long half-life of the TTL protein. Although the developmental arrest of projecting neurons was already observed at E16/E17, a reduced amount of tyrosinated α-tubulin could not be confirmed by Western blotting at this time point. These results underline the importance of TTL as even a small reduction in tyrosinated α-tubulin, which could not be detected by Western blotting, led to a strong phenotype. In a previous study, hippocampal neurons from conventional TTL knock-out embryos were shown to exhibit accelerated growth of neuronal processes in the first hours after plating (Erck et al., 2005). To investigate this *in vivo*, dendrite formation was determined in the neocortex of adult TTL^lox/lox/NexCre+^ mice. In control mice (TTL^lox/lox^), the peripheral cortex (Layers II and III) was filled with parallel-aligned bundles of microtubules decorated with microtubule-associated protein 2 (MAP-2), a marker for dendrites (Supplementary Figure 2E, the left panel). At the edge of the marginal zone, also called Exner’s plexus, MAP-2 distribution appeared more diffuse. In contrast, the TTL^lox/lox/NexCre+^ mice showed only a diffuse meshwork of MAP-2-positive structures/microtubules. Neither were oriented dendrites clearly visible, nor was the boundary between the marginal zone and the inner mass of the cortex distinct (Supplementary Figure 2E, the right panel). Thus, neurons from the TTL^lox/lox/NexCre+^ mice showed altered maturation, and dendrites failed to develop properly *in vivo* in the absence of TTL.

### Increased anxiety and impaired spatial learning in mice lacking tubulin-tyrosine ligase in the neocortex and hippocampus

**FIGURE 2:**
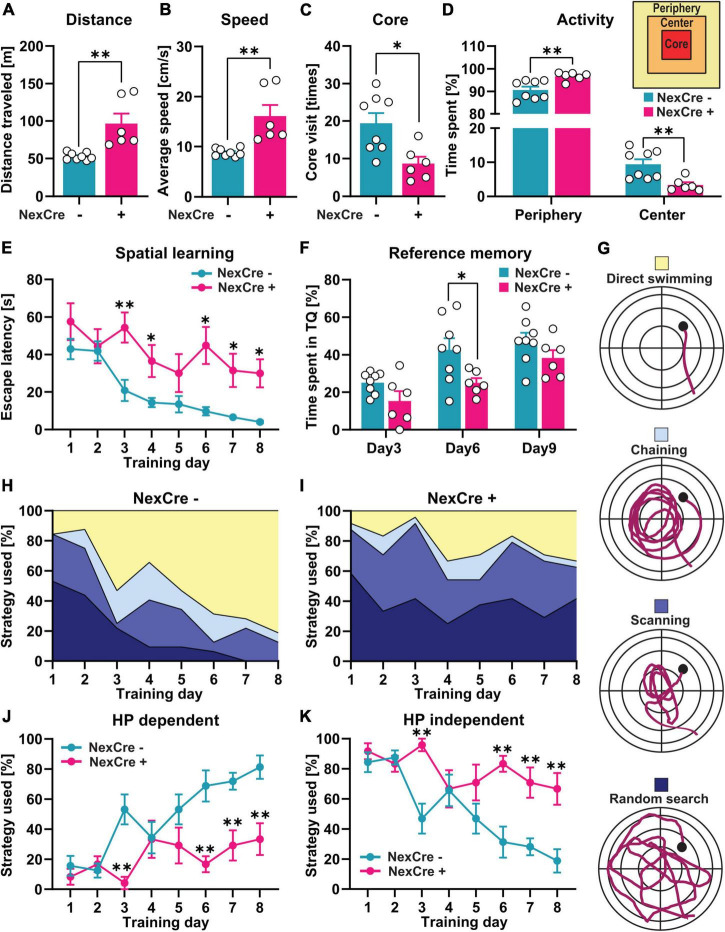
Absence of TTL in the neocortex and hippocampus results in impaired cognitive function. **(A–D)** 4–5-month-old TTL^lox/lox/NexCre+^ mice compared with control mice traveled a longer distance **(A)**, had a higher average speed **(B)**, a lower number of core visits **(C)**, and spent more time in the periphery and less time in the center zones of the open-field arena **(D)**. **(E,F)** In the Morris water test, increased escape latency **(E)** and lower percent time spent in the target quadrant **(F)** indicate impaired spatial learning and memory formation in the TTL^lox/lox/NexCre+^ mice compared with the control mice. **(G–K)** Analysis of learning strategies used during water maze training **(G)** showed that the TTL^lox/lox/NexCre+^ mice used a less hippocampus-dependent search strategy and more hippocampus-independent search strategies to find the hidden platform compared with the control mice during 8 days of learning **(H–K)**. Data are presented as mean ± SEM, *p < 0.05 and ^**^p < 0.01, *N* = 6–8 in each group.

Recent evidence has shown that microtubules are highly dynamic and play an important role in memory formation. In particular, learning is associated with changes in microtubule turnover and stability, as pharmacological manipulations of microtubule dynamics alter learning and memory processes (Uchida and Shumyatsky, 2015). To investigate the importance of α-tubulin tyrosination for hippocampal function, general locomotor activity and anxiety-related behavior as well as spatial learning were examined in conditional TTL KO mice (Figure 2).

The TTL^lox/lox/NexCre+^ mice traveled a significantly longer distance (96.42 ± 13.38 m) with a higher average speed (16.07 ± 2.22 cm/s) compared to the control mice in the open field test (distance: 52.95 ± 1.70 m, speed: 8.82 ± 0.28 cm/s) (unpaired *t*-test: *t* = 3.75, df = 12, *p* = 0.002, Figures 2A,B). The TTL^lox/lox/NexCre+^ mice showed less visits to the core area of the open-field arena (NexCre-: 19 ± 3 times, NexCre + : 9 ± 2 times, unpaired *t*-test: *t* = 3.03, df = 12, *p* = 0.01, Figure 2C) and spent less time in the center and more time in the periphery [two-way ANOVA: *F*_interaction_ (1, 24) = 21.64, *p* = 0.001, Figure 2D]. These results indicate hyperactivity and increased anxiety-related behavior in the TTL^lox/lox/NexCre+^ mice compared with the littermate controls.

To investigate spatial learning, the mice were trained in the Morris water maze for 8 days. Here, the latency to reach a hidden platform was analyzed to assess spatial learning on consecutive days. As shown in Figure 2E, the swimming time to reach the platform decreased over the course of the 8-day acquisition training in control animals [one-way RM ANOVA *F*_(7,49)_ = 16.24, *p* < 0.0001], but not in the TTL^lox/lox/NexCre+^ mice [one-way RM ANOVA *F*_(7,35)_ = 1.80, *p* = 0.12], indicating an impairment in learning. A daily comparison between the two groups showed that the escape latency was significantly higher in the TTL^lox/lox/NexCre+^ animals than in the control mice during the different training days [two-way RM ANOVA *F*_Genotype_ (1, 12) = 23, *p* = 0.0004]. To assess spatial reference memory, the animals were subjected to probe trials without the platform on Days 3 and 6 before the training and 24 h after the last training session on Day 9. The TTL^lox/lox/NexCre+^ mice spent less time in the target quadrant (TQ) compared to the control animals [two-way RM ANOVA *F*_Genotype_ (1, 12) = 10.32, *p* = 0.0075], which was significant at Day 6 (*p* = 0.026, Figure 2F). Learning impairment can be further revealed by analyzing search strategies during the training (Figures 2G–K). Based on the time spent in the different zones of the pool, including Wishaw’s corridor (direct swimming), a donut-shaped zone (chaining), a central zone (scanning), and the entire area of the pool (random search), the search strategies used by the animals can be divided into (i) a hippocampus-dependent, allocentric strategy (direct swimming, suggesting the formation of a spatial memory) and (ii) hippocampus-independent, egocentric strategies (lack of spatial memory formation), including chaining, scanning, and random search (Figure 2G). Over time, mice learn to use the more efficient allocentric strategy to navigate to the hidden platform (Garthe et al., 2009; Garthe and Kempermann, 2013). The results showed that the TTL^lox/lox/NexCre+^ mice failed to switch from the hippocampus-independent strategies to the hippocampus-dependent strategy during the training period (Figures 2H,I). Further quantification showed that the TTL^lox/lox/NexCre+^ mice were significantly less likely to use the hippocampus-dependent strategy (Figure 2J) and more likely to use the hippocampus-independent strategies (Figure 2K) on Days 3, 6, 7, and 8 of the training compared to the control mice [two-way RM ANOVA *F*_Genotype_ (1, 12) = 18.41, *p* = 0.001]. Overall, these results show a severe impairment in learning in TTL^lox/lox/NexCre+^ mice.

### Impaired hippocampal long-term synaptic plasticity and reduced spine density in forebrain-specific tubulin-tyrosine ligase knockout mice

**FIGURE 3:**
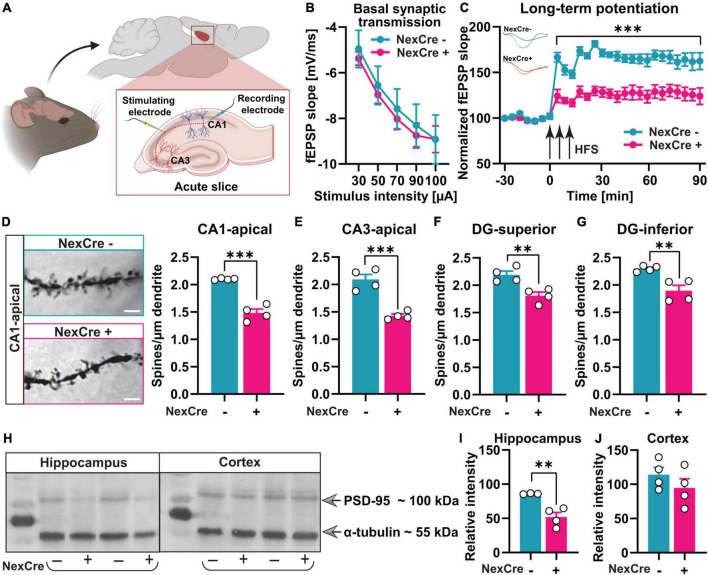
Absence of TTL in neocortex and hippocampus results in impaired synaptic plasticity and reduced density of dendritic spines. **(A)** A schematic overview of the location of stimulating and recording electrodes in acute hippocampal slices (the figure was created using BioRender.com). **(B)** Input-output curves of field excitatory postsynaptic potential (fEPSP) slopes in 7–10 hippocampal slices obtained from both tested groups did not show any significant differences. **(C)** LTP measurements in acute hippocampal slices from 4 to 5-month-old TTL^lox/lox/NexCre+^ and control mice showed severe LTP impairment. FEPSPs (normalized to baseline values) from 7 to 10 slices each of the TTL^lox/lox/NexCre+^ and control mice before and after high-frequency stimulation (HFS) are shown. **(D–G)** Brains of the 4–5-month-old TTL^lox/lox/NexCre+^ and control mice were subjected to Golgi-Cox staining, and dendritic spines in hippocampal neurons were counted. Representative examples of dendritic spines in CA1 hippocampal neurons are shown for each group (63×); a scale bar = 2 μm **(D)**. The density of dendritic spines in TTL^lox/lox/NexCre+^ was lower in the apical dendrites of CA1 **(D)** and CA3 neurons **(E)**, and in granule cells in the superior **(F)** and inferior **(G)** blades of the dentate gyrus compared with the control mice. In each group, 4 mice and 10 dendrites per mouse were analyzed and averaged. **(H)** Western blot analysis of hippocampus and cortex lysates from the 10-week-old mice showed that **(I)** PSD-95 levels were reduced in hippocampi of the TTL^lox/lox/NexCre+^ mice compared with the control mice **(J)**, and this was not the case in the cortex. The relative intensities of the signals were measured by densitometry and normalized to total α-tubulin (*n* = 3–4). Data are presented as mean ± SEM, ^**^p < 0.01 and ^***^p < 0.001.

To further investigate the cellular basis for the observed memory impairment in the TTL^lox/lox/NexCre+^ mice we analyzed long-term potentiation (LTP). For this purpose, acute hippocampal slices from 4- to 5-month-old control mice and TTL^lox/lox/NexCre+^ mice were prepared. LTP was triggered by three stimulus trains (HFS) at the Schaffer collateral/commissural CA3-CA1 pathway, and field excitatory postsynaptic potentials (fEPSP) were recorded in the stratum radiatum of the CA1 hippocampal region (Figure 3A). Before LTP assessment, to evaluate changes in basal synaptic transmission, the dependence of the slope of fEPSPs on stimulation intensity was analyzed in input/output curves (Figure 3B). No differences were found between experimental groups [two-way RM ANOVA *F*_(1,15)_ = 0.09, *p* = 0.76, Figure 3B]. This result suggests that basal synaptic transmission was not affected by the deletion of TTL during development. After baseline recording, LTP was induced and strong potentiation was observed in the hippocampus of the control mice. However, in the TTL^lox/lox/NexCre+^ mice, LTP was severely impaired (Figure 3C), indicating perturbed hippocampal networks.

As a next step, spine density was analyzed by the Golgi-Cox method in the TTL^lox/lox/NexCre+^ and control mice. Changes in dendritic spine density and morphology, indeed, have been shown to correlate with defects in synaptic plasticity and cognitive function in general (Moser et al., 1994). After deletion of TTL, dendritic spine density was reduced in CA1 (Δ29.5%, unpaired *t*-test: *t* = 8.65, df = 6, *p* = 0.0001, Figure 3D) and CA3 (Δ31.8%, unpaired *t*-test: *t* = 6.50, df = 6, *p* = 0.0006, Figure 3E), consistent with the loss of tyrosinated α-tubulin, particularly in hippocampal pyramidal cells (Supplementary Figures 1A,B). Further analysis revealed significantly decreased dendritic spine density in granule cells, which was evident in both suprapyramidal (Δ17.4%, unpaired *t*-test: *t* = 4.05, df = 6, *p* = 0.006, Figure 3F) and infrapyramidal (Δ17.5%, unpaired *t*-test: *t* = 3.97, df = 6, *p* = 0.007, Figure 3G) blades of the dentate gyrus in the TTL^lox/lox/NexCre+^ mice compared to the controls. Normal levels of tyrosinated α-tubulin were observed in the dentate gyrus, likely due to transient expression of Cre recombinase over time in this area (Goebbels et al., 2006; Supplementary Figure 1A, the arrow). Hence, impaired network formation in cortical input areas of the hippocampus as well as in hippocampal pyramidal cells might negatively affect the entire hippocampal network and, consequently, the granule cells in the dentate gyrus region.

In the dentate gyrus, synaptic contacts are predominantly formed by different systems of afferents, the entorhinal and commissural-associational fibers. It is suggested that a reduction in these afferent fibers leads to a reduction in postsynaptic compartments on dentate gyrus neurons (Geinisman et al., 1992).

The reduced spine density in the absence of TTL suggests a role of TTL in synaptogenesis. Therefore, postsynaptic density protein 95 (PSD-95), a marker of excitatory postsynaptic boutons, was analyzed as well and found to be less abundant in hippocampal lysates from the TTL^lox/lox/NexCre+^ mice (NexCre-: 85.97 ± 0.43%, NexCre+: 51.80 ± 6.74%, unpaired *t*-test: *t* = 4.28, df = 5, *p* = 0.008, Figures 3H,I). This was not the case in the neocortex (NexCre-: 114.1 ± 10.47%, NexCre+: 94.5 ± 13.63%, unpaired *t*-test: *t* = 1.14, df = 6, *p* = 0.29, Figures 3H,J), indicating a role of TTL more for axonal outgrowth in this region. In summary, deletion of TTL in the hippocampus leads to impaired synapse formation as well as defects in synaptic plasticity and spatial learning.

### CA1 pyramidal cell-specific tubulin-tyrosine ligase deletion in the hippocampus reveals an acute role for synaptic plasticity

**FIGURE 4:**
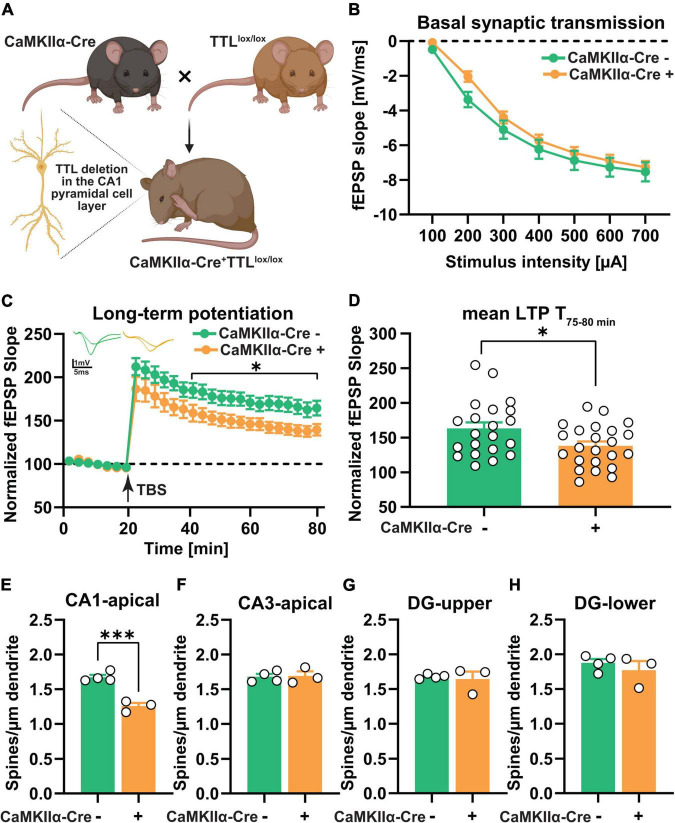
CA1-specific TTL deletion in the hippocampus results in impaired long-term synaptic plasticity. **(A)** Schematic representation of TTL^lox/lox/CaMKIIα–Cre+^ mouse generation (the figure was created using BioRender.com). **(B)** Input-output curves of field excitatory postsynaptic potential (fEPSP) slopes in hippocampal slices obtained from both tested groups did not show any significant differences. **(C)** Acute hippocampal slices from the TTL^lox/lox/CaMKIIα–Cre+^ mice showed significantly lower LTP compared with the control mice. FEPSPs (normalized to baseline values) of 21–23 slices each from the TTL^lox/lox/CaMKIIα–Cre+^ and control mice before and after θ-burst stimulation (TBS) are shown **(C)**. The TTL^lox/lox/CaMKIIα–Cre+^ mice showed significantly reduced maintenance (T, 75–80 min) of LTP compared with the control mice **(D)**. **(E–H)** Dendritic spine density in CA1 pyramidal neurons was lower in the TTL^lox/lox/CaMKIIα–Cre+^ mice compared with the control mice **(E)**, whereas, dendritic spine density in CA3 neurons **(F)** and granule cells located in the superior **(G)**, and inferior **(H)** blades of the dentate gyrus showed no significant differences. In each group, 3–4 mice and 10 dendrites per mouse were analyzed and averaged. Data are presented as mean ± SEM, *p < 0.05 and ^***^p < 0.001.

The results presented so far showed that tyrosination of α-tubulin in the neocortex and hippocampus during development is important for network formation and that forebrain-specific deletion of TTL, therefore, leads to cognitive deficits and impaired synaptic plasticity. In a next step, we were, therefore, interested in whether TTL is also acutely involved in synaptic plasticity processes underlying learning and memory formation in mature neurons. For this purpose, we crossed the TTL^lox/lox^ mice with a specific calcium-calmodulin kinase II (CaMKII)α-Cre reporter mouse line [Tg(Camk2a-cre)T29-1Stl, Figure 4A]. Cre-dependent lacZ reporter expression in this line predominantly showed labeling of the CA1 pyramidal cell layer in the hippocampus, starting during the 3rd to 4th postnatal weeks (Tsien et al., 1996). By this means, developmental defects can be prevented. The TTL^lox/lox/CaMKIIα–Cre+^ mice were born with a normal Mendelian ratio, were healthy, and fertile. A previous study has shown that target gene deletion was nearly complete at 3 months of age in the hippocampus of CaMKIIα-Cre + mice (Chen et al., 2017). To ensure that the TTL deletion was complete in mature CA1 pyramidal neurons, adult animals at 4–5 months of age were used in this study.

Prior to the assessment of long-term plasticity, the input/output curves were analyzed to reveal potential deficits in basal synaptic transmission (Figure 4B). The results showed no significant differences between the two groups, indicating that basal synaptic transmission was not affected in the absence of TTL, specifically in CA1 pyramidal neurons [two-way RM ANOVA *F*_(1,40)_ = 1.03, *p* = 0.31, Figure 4B]. In a next step, to assess synaptic plasticity, LTP was examined in the TTL^lox/lox/CaMKIIα–Cre+^ and control mice (Figures 4C,D). For this purpose, after a 20-min baseline recording, θ-burst stimulation (TBS) was used to induce LTP at the Schaffer collateral/commissural CA3-CA1 pathway in acute slices from the TTL^lox/lox/CaMKIIα–Cre+^ and control mice. Control slices showed robust induction of LTP and a sustained maintenance phase (Figure 4C). In contrast, the TTL^lox/lox/CaMKIIα–Cre+^ slices showed a significant reduction [two-way RM ANOVA *F*_Genotype_ (1, 42) = 5.50, *p* = 0.02, Figure 4C], especially in the maintenance phase of LTP, as shown by the mean value of LTP in the last 5 min of recording (163.2 ± 8.7% in the control slices vs. 138.1 ± 6.3% in the mutant sections, unpaired *t*-test: *t* = 2.36, df = 42, *p* = 0.02, Figure 4D). The impairment in LTP observed in the TTL^lox/lox/CaMKIIα–Cre+^ mice suggests that postsynaptic plasticity in the mature hippocampus is acutely compromised by loss of TTL.

To investigate the possible cellular mechanism underlying the observed LTP deficit in the TTL^lox/lox/CaMKIIα–Cre+^ mice, the morphology of neurons in the hippocampus was analyzed by Golgi-Cox staining (Figures 4E–H). As mentioned above, changes in the density of dendritic spines have been shown to correlate with defects in LTP (Moser et al., 1994). Consistent with the predominantly CA1-specific deletion of TTL, only the number of dendritic spines on CA1 pyramidal neurons in the hippocampus of the TTL^lox/lox/CaMKIIα–Cre+^ mice showed a significant reduction compared with the control mice (Δ25%, unpaired *t*-test: *t* = 7.60, df = 5, *p* = 0.0006, Figure 4E). Dendritic spines in the other hippocampal subregions, including CA3 and dentate gyrus, did not show significant differences compared to the control mice (CA3: Δ0.65%, unpaired *t*-test: *t* = 0.15, df = 5, *p* = 0.88, Figure 4F; DG-superior: Δ2.20%, unpaired *t*-test: *t* = 0.39, df = 5, *p* = 0.70, Figure 4G; DG-inferior: Δ5.60, unpaired *t*-test: *t* = 0.81, df = 5, *p* = 0.45, Figure 4H).

**FIGURE 5:**
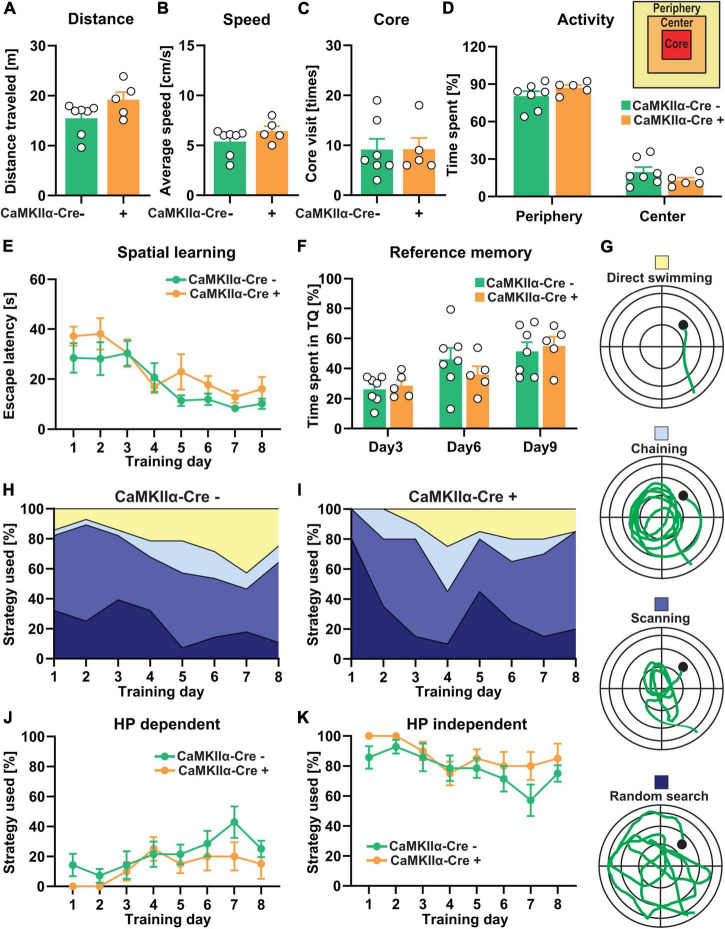
CA1-specific TTL deletion in the hippocampus does not lead to severe impairment of cognitive functions. **(A–D)** Total distance traveled **(A)**, average speed **(B)**, number of core visits **(C)**, and percentage of activity of mice in the periphery and the center of the open-field arena **(D)** showed no significant differences between the TTL^lox/lox/CaMKIIα–Cre+^ and control mice. **(E–K)** In the Morris water maze test, during the 8-day acquisition training, escape latency significantly decreased in both the TTL^lox/lox/CaMKIIα–Cre+^ and control groups, indicating spatial learning, but it was not significantly changed between the groups **(E)**. **(F)** The percentage of time the TTL^lox/lox/CaMKIIα–Cre+^ and control mice spent in the target quadrant did not show significant differences. **(G)** Different search strategies, including hippocampus-dependent (direct swimming) and hippocampus-independent (random search, scanning, and chaining), were presented and color-coded **(H,I)**. The TTL^lox/lox/CaMKIIα–Cre+^ mice used slightly less hippocampus-dependent **(J)** and more hippocampus-independent **(K)** search strategies compared with the control mice. Data are presented as mean ± SEM, *N* = 5–7 in each group.

The TTL knockout mice (TTL^lox/lox/CaMKIIα–Cre+^) were also behaviorally characterized. This was first done in the open-field arena to examine general locomotor activity and assess anxiety-related behavior (Figures 5A–D). Analysis of total distance traveled (Figure 5A), average speed (Figure 5B), number of visits to the core area (Figure 5C), and overall activity and time spent in the peripheral and central zones of the arena (Figure 5D) revealed no significant differences between the TTL^lox/lox/CaMKIIα–Cre+^ mice and the control littermates [distance, Cre-: 15.45 ± 1.21 m, Cre + : 19.17 ± 1.54 m; unpaired *t*-test: *t* = 1.92, df = 10, *p* = 0.08, Figure 5A; speed, Cre-: 5.38 ± 0.5 cm/s, Cre + : 6.42 ± 0.5 cm/s; unpaired *t*-test: *t* = 1.41, df = 10, *p* = 0.19, Figure 5B; core visit: Cre-: 9 ± 2 times, Cre + : 9 ± 2 times; unpaired *t*-test: *t* = 0.018, df = 10, *p* = 0.98, Figure 5C; activity in the periphery and the center: two-way ANOVA *F*_Genotype_ (1, 20) = 0.0001, *p* > 0.99, Figure 5D]. These results show that deletion of TTL in mature CA1 pyramidal neurons alone (TTL^lox/lox/CaMKIIα–Cre+^) does not alter general behavior as it was observed for the forebrain-specific deletion (TTL^lox/lox/NexCre+^).

Previous findings on the involvement of the hippocampus in learning and memory have suggested that different cortical regions and hippocampal CA fields subserve important roles (Suthana et al., 2009). Therefore, the TTL^lox/lox/CaMKIIα–Cre+^ mice were trained in the Morris water maze (Figures 5E–K). In contrast to the TTL^lox/lox/NexCre+^ mice, swimming time to reach the hidden platform significantly decreased in both the TTL^lox/lox/CaMKIIα–Cre+^ [one-way RM ANOVA *F*_(7,28)_ = 4.34, *p* = 0.002] and control mice [one-way RM ANOVA *F*_(7,42)_ = 4.66, *p* = 0.0006] during the 8-day acquisition training. The learning performance of the TTL^lox/lox/CaMKIIα–Cre+^ mice did not differ from the control animals [two-way RM ANOVA *F*_Genotype_ (1, 10) = 4.58, *p* = 0.057, Figure 5E]. The reference memory tests on Days 3, 6, and 9 showed also no significant differences in time spent in the target quadrant between the TTL^lox/lox/CaMKIIα–Cre+^ and control mice [two-way RM ANOVA *F*_Genotype_ (1, 10) = 0.042, *p* = 0.84, Figure 5F]. Evaluation of the respective search strategies showed that the hippocampus-dependent search strategy increased over time, whereas, the hippocampus-independent search strategies decreased in both the control and TTL^lox/lox/CaMKIIα–Cre+^ mice (Figures 5G–I). However, the TTL^lox/lox/CaMKIIα–Cre+^ mice showed a slight reduction in the usage of hippocampus-dependent strategies (Figure 5J) and, instead, used more the hippocampus-independent strategies (Figure 5K) compared with the control animals; however, this was not significant [two-way RM ANOVA *F*_Genotype_ (1, 10) = 3.05, *p* = 0.11]. These results suggest that the specific deletion of α-tubulin tyrosination in mature CA1 pyramidal neurons does not lead to severe impairments in spatial learning, in contrast to observations in the TTL^lox/lox/NexCre+^ mice, in which abnormalities of the entire brain network, including incomplete development of the corpus callosum and anterior commissures due to axonal growth arrest, can be observed.

### Deletion of tubulin-tyrosine ligase in single cells in hippocampal slice cultures shows a crucial role for activity-dependent spine structural plasticity

**FIGURE 6:**
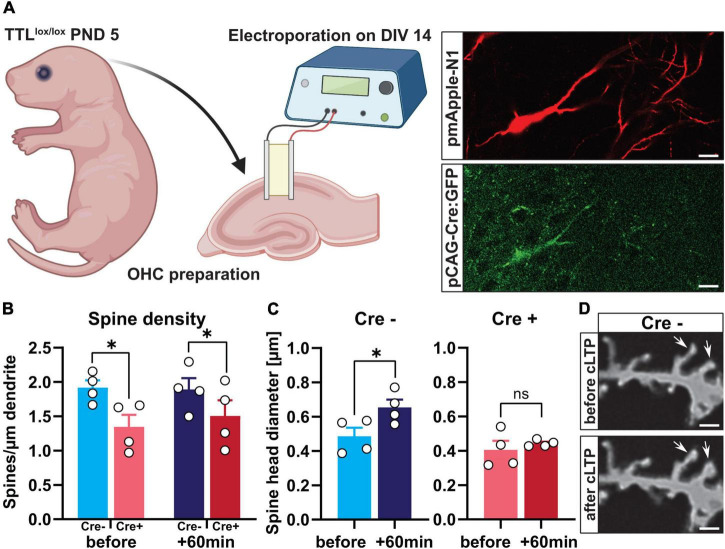
Single-cell deletion of TTL in hippocampal slice cultures impairs activity-dependent structural plasticity. **(A)** Schematic representation of single-cell deletion of TTL in hippocampal slice cultures derived from TTL^lox/lox^ mice at postnatal Day 5. Cells were transfected with pmApple-N1 and pCAG-Cre:GFP plasmids by single-cell electroporation (a scale bar = 20 μm) (the figure was generated using BioRender.com). **(B)** Assessment of dendritic spine density showed that acute depletion of TTL in Cre + cells resulted in a significant reduction in the number of dendritic spines compared with control Cre- cells, but cLTP induction did not trigger new spine formation (*N* = 4 in each group). **(C,D)** Sixty minutes after cLTP induction, Cre- control neurons showed a significant increase in a spine-head diameter, which was not the case in Cre + cells lacking TTL **(C)**. Representative examples of dendritic spines in Cre- control cells before and after cLTP induction are shown (60×); scale = 2 μm **(D)**. *N* = 4 cells in each group and 15 spines along each dendrite were randomly selected, measured, and averaged for head diameter assessment. Data are presented as mean ± SEM, *p < 0.05.

To even further characterize the acute role of TTL for synaptic plasticity at the single-cell level, we used TTL deletion in organotypic hippocampal slice cultures. Slice cultures (OHCs) were obtained from the TTL^lox/lox^ mice at postnatal Day 5 and then transfected with pmApple-N1 and pCAG-Cre:GFP plasmids at days *in vitro* (DIV) 14 using single-cell electroporation as described previously (Michaelsen-Preusse et al., 2014). By this means, we were able to avoid defects resulting from improper synapse development (Figure 6A). Electroporation of single cells with the pCAG-Cre:GFP plasmid resulted in expression of Cre recombinase fused to GFP in TTL^lox/lox^ cells in OHCs. Further transfection of the cells with the pmApple-N1 plasmid allowed detailed visualization of neuronal structure. The cells expressing only mApple were used as controls (Cre-) (Figure 6A). To elucidate the role of α-tubulin tyrosination in activity-dependent structural plasticity of dendritic spines, NMDAR-dependent LTP was induced by administration of 10-mM glycine for 10 min and 4 days after transfection (chemical LTP, cLTP). Glycine-induced cLTP is reported to result in a significant increase in synaptic efficacy that is comparable in many aspects to the long-term potentiation induced by θ-burst stimulation (Fortin et al., 2010; Michaelsen-Preusse et al., 2016). Spines located at the proximal apical dendrites of pyramidal neurons were imaged before and 60 min after cLTP induction. Assessment of dendritic spine density showed that, in line with our *in vivo* data, also, acute depletion of TTL in Cre + cells resulted in a significant reduction in the number of dendritic spines compared with control Cre- cells [two-way ANOVA *F*_Genotype_ (1, 12) = 7.61, *p* = 0.01, Figure 6B]. In both control and TTL-depleted single neurons, cLTP induction did not trigger new spine formation, as the number of dendritic spines before and 60 min after cLTP was comparable in each group [two-way ANOVA *F*_*Time after cLTP*_ (1, 12) = 0.15, *p* = 0.70, Figure 6B]. In line with the results described above, indicating a role for TTL in plasticity processes, we found that spine structural plasticity was disrupted in TTL-deficient neurons (Figure 6C). Sixty min after cLTP induction, Cre- control neurons showed a significant increase in a spine head diameter (unpaired *t*-test: *t* = 2.48, df = 6, *p* = 0.04, Figures 6C,D). In contrast, positive spine structural plasticity was not detectable in the absence of TTL (unpaired *t*-test: *t* = 0.70, df = 6, *p* = 0.50, Figure 6C). These experiments demonstrate that tyrosination of α-tubulin at the postsynapse is important for structural plasticity processes.

## Discussion

Microtubules have pleiotropic roles in various cellular processes, and posttranslational modification (PTMs) of tubulin is one of the main factors modulating their diverse function as it results in a specific microtubule surface pattern known as the “tubulin code.” Yet, the detailed physiological role of this code remains poorly understood (Janke and Bulinski, 2011; Janke and Magiera, 2020). The first tubulin PTM was described as an RNA-independent enzymatic incorporation of tyrosine observed mainly in neuronal cells (Song et al., 2015). In neurons, both detyrosinated and tyrosinated tubulins have been shown to be distributed in patches along axonal MTs, with detyrosinated tubulins enriched in proximal segments of axon shafts and tyrosinated ones in growth cones (Brown et al., 1993). Detyrosinated MTs are resistant to degradation, whereas tyrosination of α-tubulin has been defined as a marker for dynamic MTs. Indeed, the distal regions of neurites are predominantly tyrosinated (Moutin et al., 2021). In this study, we were interested whether this modification might be important for development but, moreover, also, for neuronal plasticity processes. A crucial role during early development was suggested by our previous results in TTL-null mice that died within 24 h of birth with respiratory problems, ataxia, and severely altered neuronal morphologies (Erck et al., 2005). It was later determined that the severe perinatal mortality in the TTL-null mice may be related to alterations in neurite formation, axon guidance, and increased Rac1 signaling (Marcos et al., 2009). We now aimed to investigate the detailed role of detyrosination/tyrosination PTM both during forebrain development and in mature neurons using conditional deletion of TTL at different time points and in different brain regions.

Tubulin-tyrosine ligase deletion in post mitotic glutamatergic projection neurons in the neocortex and hippocampus exploiting the Nex promoter resulted in pronounced effects on network formation. The development of the cortico-thalamic loop appeared to be reduced, but correct synaptic connections were formed between the TTL-deficient cortical neurons and the TTL-positive-projecting thalamic neurons. This suggests that, although deletion of the TTL gene by NexCre begins at E11.5 (Goebbels et al., 2006), the effects on growth of cortical or thalamic axons, which begins at E13 (López-Bendito and Molnár, 2003), are limited. On the other hand, we defined a developmental arrest at E16/E17, manifested by reduced formation of projecting axons in the developing corpus callosum and ventral commissures. Remarkably, using Western blot analyses, we observed a reduction in α-tubulin tyrosination only from postnatal Day 7. Thus, we conclude that cortical neurons forming the cortico-thalamic loop or callosal projections, such as the corpus callosum, begin projecting before TTL protein is completely absent and that cortical neurons forming the ventral commissures later in development do not project because of the complete loss of TTL at this time point. Previously, it has been shown that *in vitro* isolated TTL-deficient hippocampal neurons initially grow faster, develop multiple axons, and are more branched (Erck et al., 2005). This is in marked contrast to the *in vivo* data, where normal axon formation, e.g., by the corpus callosum in both hemispheres in the TTL^lox/lox/NexCre+^ mice, was drastically reduced. Apparently, suppression of TTL disrupted mechanisms essential for controlling neurite outgrowth, but not neurite outgrowth *per se* (Erck et al., 2005). Furthermore, previous findings showed that initiation of neurite outgrowth *in vitro* is generally not dependent on microtubule assembly. In neuronal cultures treated with taxol, processes formed despite inhibition of the microtubule assembly, maintenance of stable acetylated alpha-tubulin, and the absence of dynamic tyrosinated alpha-tubulin. These results suggest that the initial steps of neurite formation do not depend on the microtubule assembly and suggest that microtubules assembled in the cell body may be translocated to developing neurites as they form (Smith, 1994). However, further experiments are needed to fully uncover the underlying mechanism.

Behavioral analysis showed that the TTL^lox/lox/NexCre+^ mice exhibited hyperactivity and anxiety-related behaviors. Indeed, healthy neuronal function and proper network formation are crucial events based on normal cytoskeletal organization and dendritic branching. Immunoreactivity of microtubule-associated protein 2 (MAP-2) has been shown to be reduced in the hippocampus and prefrontal cortex of postmortem tissue from patients with schizophrenia (Shelton et al., 2015). Moreover, several features of mouse models associated with schizophrenia, such as anxiety, cognitive deficits, hyperactivity, social impairments, and glutamatergic transmission, are due to genetic deletion of MAPs (Bonini et al., 2017). Additionally, a knock-out model for MAP1B resembles the phenotype of the conventional TTL knock-out mouse in terms of impaired formation of the corpus callosum, thalamo-cortical loop, and hippocampal and anterior commissures (Del RíO et al., 2004). These observations support the idea that tyrosinated α-tubulin is a downstream target for phosphorylated MAPs. Our results also demonstrate the diffuse and reduced immunoreactivity of MAP-2 in the neocortex of TTL^lox/lox/NexCre+^ mice. This underscores previous findings that attention deficits and hyperactivity disorders may be due to delayed establishment of corticolimbic circuitry or impaired dopamine neurotransmission (Drerup et al., 2010).

Moreover, stathmin has been shown to affect microtubule dynamics by promoting microtubule depolymerization or preventing polymerization of tubulin heterodimers, potentially regulating anxiety, fear, and learning (Uchida et al., 2014; Uchida and Shumyatsky, 2015). STMN1 KO mice exhibited anxious hyperactivity, impaired object recognition, and lower levels of neutral and social exploratory behavior at the baseline compared with wild-type mice (Nguyen et al., 2019). This suggests that changes in microtubule dynamics due to inhibition of the post-translational modifications may trigger a hyperactivity phenotype.

In addition to hyperactivity, the TTL^lox/lox/NexCre+^ mice showed severe impairment in spatial learning and decreased use of hippocampus-dependent search strategies in the water maze test. It has been shown that the synaptic events required for the consolidation of memory traces in cortical networks require time-dependent, coordinated, and correct hippocampal-cortical interactions, but the underlying mechanisms remain unclear (Frankland et al., 2001; Lesburguères et al., 2011). Furthermore, decreased expression of MAP-2 and resulting dystrophic neurites can be found associated with impaired learning and memory formation in Alzheimer’s disease models (Ma et al., 2014). Apart from the maldevelopment of hippocampal and cortical circuits in TTL^lox/lox/NexCre+^ mice, which are most likely responsible for the observed deficits in spatial memory formation, the proper transport of organelles and synaptic components, including vesicles containing glutamate receptor subunits and other cargoes, to postsynaptic sites along MTs crucial for learning and memory formation (Setou et al., 2000) might be impaired in these mice.

In line with the severe deficits in spatial learning in forebrain-specific TTL knock-out mice, our results showed that long-term synaptic plasticity was also severely impaired as indicated by decreased LTP and reduced dendritic spine density after deletion of TTL in TTL^lox/lox/NexCre+^ mice. Basal synaptic transmission was not affected, which might point toward potentially compensatory mechanisms due to chronic loss of TTL, leading to a normal steady-state level of synaptic ligand-gated ion channel expression.

Furthermore, our results showed that PSD-95 levels were decreased in the neocortex and hippocampus of these mice. In general, three groups of proteins have been described that can discriminate between microtubules enriched in either tyrosinated or detyrosinated α-tubulin: (i) microtubule + TIP-binding proteins (Steinmetz and Akhmanova, 2008), (ii) microtubule-destabilizing proteins (Peris et al., 2009), and (iii) molecular motor proteins (Konishi and Setou, 2009). Of these, the microtubule + end-binding protein EB3 regulates dendritic morphology by directly interacting with PSD-95, and local accumulation of PSD-95 in spine heads directs dynamic microtubules into these spines (Hu et al., 2011). It can be hypothesized that a large pool of stable detyrosinated microtubules is present in the dendrites of TTL-deficient neurons and that highly motile microtubules, which can enter spines during the induction of synaptic plasticity, for instance, are sparse or completely absent. This may explain the observed reduction in PSD-95 protein levels in TTL^lox/lox/NexCre+^ mice and the resulting lower density of dendritic spines, as well as the impaired LTP after deletion of TTL.

The results obtained from the TTL^lox/lox/NexCre+^ mice revealed the importance of α-tubulin tyrosination for the proper formation of the cortical-hippocampal network. To also understand the acute role of this PTM in synaptic plasticity, we used the T29-1 CaMKIIα-Cre mouse line to specifically remove TTL from the CA1 pyramidal cell layer of the hippocampus in the third to fourth postnatal weeks, and, therefore, after networks are already established (Tsien et al., 1996). Intriguingly, CA1-specific deletion of TTL in the TTL^lox/lox/CaMKIIα–Cre+^ mice also resulted in impaired Schaffer-collateral LTP and decreased spine density in hippocampal CA1 neurons, indicating a role also for synaptic integrity and synaptic plasticity in the adult brain. It can be concluded that the tyrosination status of α-tubulin is not only important for brain development but also plays an essential role in basal brain function as well as plasticity processes in the adult CNS.

In contrast to TTL^lox/lox/NexCre+^ mice, in TTL^lox/lox/CaMKIIα–Cre+^ mice, mainly, the maintenance phase of LTP is impaired. The specific role of tyrosination/detyrosination posttranslational modification of the microtubule for the induction and maintenance phases of LTP yet needs to be elucidated. It has been shown that stathmin is dephosphorylated in the early phase, which enhances its microtubule-destabilizing activity by promoting stathmin-tubulin binding, whereas, these processes are reversed in the late phase, leading to an increase in microtubule/KIF5-mediated localization of the GluA2 subunit of AMPA receptors to synaptic sites (Uchida and Shumyatsky, 2015). In the first 8 h after learning, changes in phosphorylation of stathmin undergo two phases that lead to biphasic shifts in microtubule stability/instability. These shifts, in turn, regulate memory formation by controlling synaptic transport of the GluA2 subunit of AMPA receptors in the second phase (Uchida and Shumyatsky, 2015). Therefore, altered microtubule stabilization in CA1 neurons could, indeed, affect the localization of the GluA2 subunit of AMPA receptors to synaptic sites, which, in turn, could affect the late phase of LTP.

For further confirmation of the importance of the tubulin codes, we additionally investigated the role of TTL in activity-dependent spine structural plasticity in hippocampal slice cultures. Indeed, loss of TTL in cultured hippocampal neurons led to an impairment of spine structural plasticity after cLTP induction. Overall, these results confirm the fact that dynamic MTs targeting spines are important for activity-dependent changes in synapse structure and function. The dynamics of MTs are necessary for the transport of cargoes required for signal transduction by kinesin and dynein along MTs to the dendrite spine head (Dent, 2017). Spine entry of dynamic microtubules is triggered by *N*-methyl-*D*-aspartic acid (NMDA) receptor activation and calcium influx and requires dynamic actin remodeling (Schätzle et al., 2018). Therefore, these results may also highlight the direct importance of α-tubulin tyrosination for actin microtubule crosstalk in orchestrating synaptic function and plasticity. Despite the impairments in functional plasticity in TTL^lox/lox/CaMKIIα–Cre+^ animals, they did not show behavioral or cognitive impairments. This might be, indeed, expected as the knock out was confined to the CA1 region only and did not alter hippocampal function in total. However, the trend toward a reduction in hippocampus-dependent search strategies that we observed in the TTL^lox/lox/CaMKIIα–Cre+^ mice is consistent with the fact that the CA1 region is strongly involved especially in the allocentric encoding of spatial information (Suthana et al., 2009).

In summary, TTL activity is a prerequisite for proper cortical development and function. Loss of TTL has been shown to be associated with an imbalance of tyrosinated and detyrosinated pools of α-tubulin resulting in reduced MT dynamics and subsequent dyslocalization of microtubule-binding proteins and arrest of neuronal growth (Erck et al., 2005; Gu et al., 2008). Our data support the finding that mutations in tubulins lead to brain malformations and can be associated with various brain disorders and diseases (Moutin et al., 2021). In fact, in neurodegenerative processes, the content of detyrosinated/tyrosinated tubulins has been shown to be altered in neurons and may be an early marker of disease development (Zhang et al., 2015). The tubulin code as generated by post-translational modifications of tubulins is thus an important intrinsic cue for proper neuronal integrity and function. We found that the α-tubulin tyrosination cycle provides the molecular and structural basis for fine-tuning neuronal connectivity and is a novel key component of synaptic plasticity and memory formation.

## Data availability statement

The raw data supporting the conclusions of this article will be made available by the authors, without undue reservation.

## Ethics statement

This animal study was reviewed and approved by Nds. Landesamt für Verbraucherschutz und Lebensmittelsicherheit, LAVES.

## Author contributions

KM-P, MH, CE, and MK conceived and designed the research. SH, MH, and CE performed the experiments. All authors analyzed the data, wrote the manuscript, and approved the submitted version.
