# Supplementary material for: The role of α-tubulin tyrosination in controlling the structure and function of hippocampal neurons
Source: Front Mol Neurosci. 2022 Oct 19;15:931859. doi: 10.3389/fnmol.2022.931859 (PMC9627282; doi:10.3389/fnmol.2022.931859)
Supplement: Supplementary file 1 [file Data_Sheet_1.docx]

Supplementary Material

## Supplementary Figures

### Supplementary Figure 1


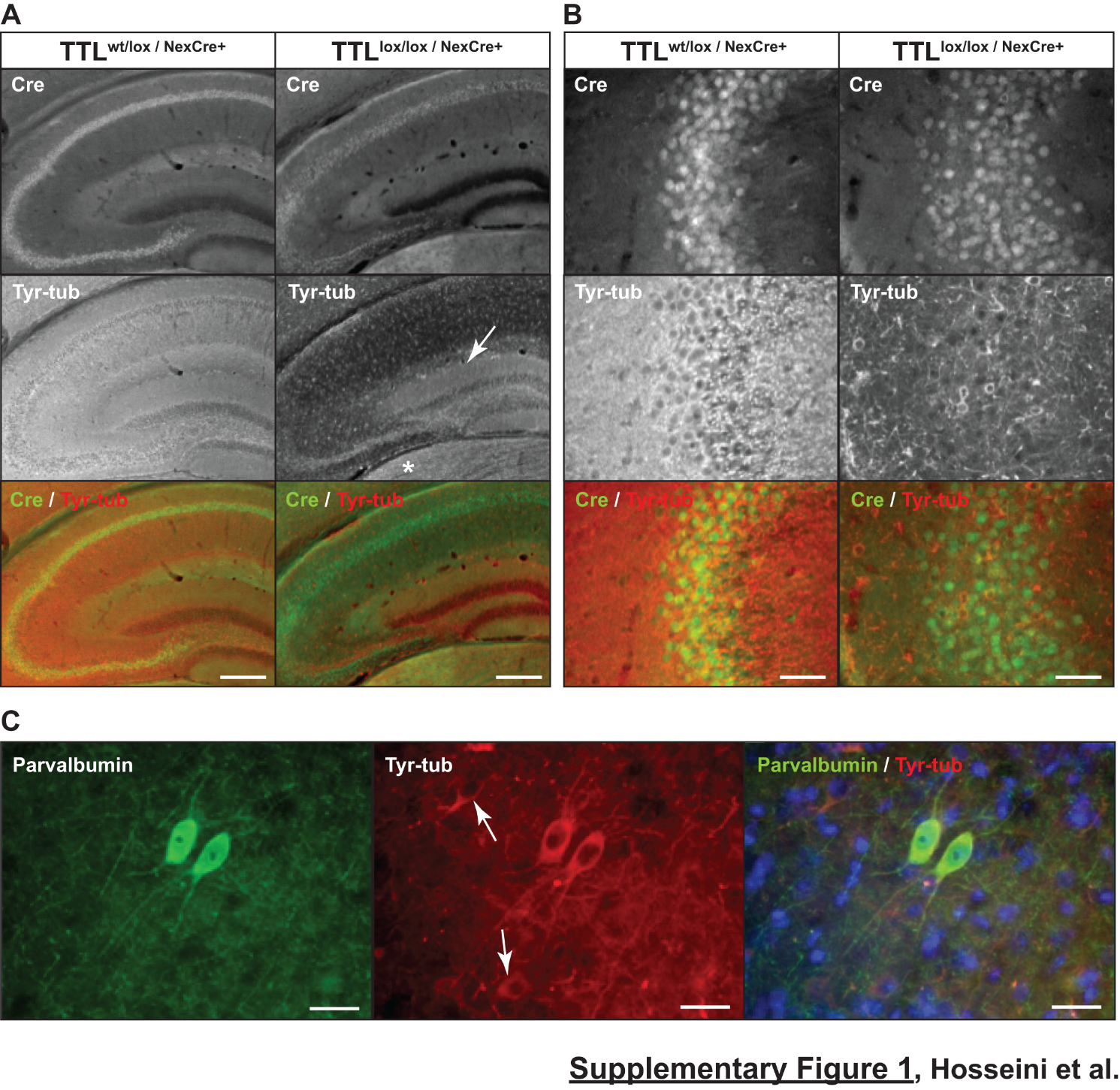
**Supplementary Figure 1 | Specificity of NexCre-mediated TTL deletion.** Immunofluorescence labeling showed loss of tyrosinated α-tubulin (Tyr-tub, red) in Cre recombinase-expressing cells (Cre, green) in the hippocampus **(A)** and at higher magnification in the hippocampal CA3 subregion **(B)** of TTL^lox/lox/NexCre+^ mice (right panels) compared with control mice (left panels). Neurons in dentate gyrus (arrow) and thalamus (asterisk) do not express Cre recombinase and are therefore rich in tyrosinated α-tubulin (scale bars are 300μm in A and 50μm in B). **(C)** Parvalbumin-positive interneurons (green) lacking Cre recombinase expression with apparently unaltered immunolabeling for tyrosinated α-tubulin (Tyr-tub, red). Tyrosinated α-tubulin is also visible in other cells that were not affected by NexCre expression (arrows). The cell nuclei are blue (DAPI) (scale bar = 20μm).

### Supplementary Figure 2

**
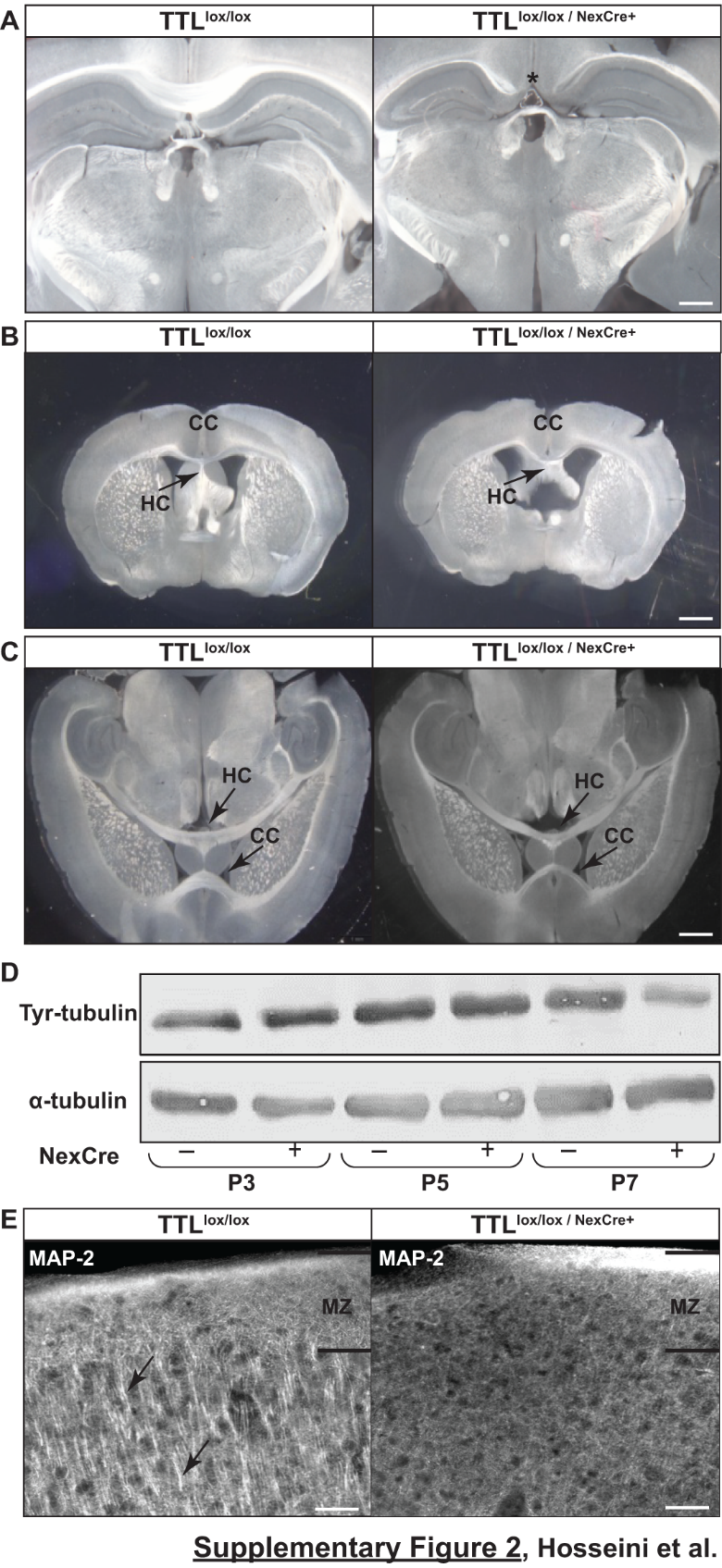
**

**Supplementary Figure 2 | Loss of TTL in the brain resulted in defects in the nerve fiber bundles. (A)** Coronal brain sections from 10-week-old TTL^lox/lox/NexCre+^ mice (right panel) showed reduced formation of the corpus callosum and incomplete midline crossing from rostral to caudal (asterisk) compared with control mice (left panel) (scale bar = 1.5mm). Coronal **(B)** and horizontal **(C)** brain sections from 10-week-old control mice (left panels) and TTL^lox/lox/NexCre+^ mice (right panels) showed multiple brain malformations, including hypoplasia of the corpus callosum (CC), reduced hippocampal commissure (HC) formation, and midline gap formation (scale bar = 1mm). **(D)** Western blot analysis of cortices from control (NexCre -) and TTL^lox/lox/NexCre+^ (NexCre +) mice at different days after birth (P3, P5, and P7) showed a decrease in tyrosinated α-tubulin only at P7. **(E)** Dendritic density analyzed with the dendritic marker microtubule-associated protein 2 (MAP2) in 10-week-old control (left panel) and TTL^lox/lox/NexCre+^ (right panel) brain slices revealed the absence of distinct MAP2-immunoreactive bundles in mice lacking TTL. MZ is the marginal zone (scale bar = 20µm).
